# Epidural cavernous haemangioma during pregnancy: a case report and a literature review

**DOI:** 10.11604/pamj.2019.33.202.8481

**Published:** 2019-07-15

**Authors:** Anas Bennis, Reda Hafiane, Jaafar Benouhoud, Amine El Khaoudi, Khadija Ibahioin, Abdelhakim Lakhdar, Ihsane Moussaid, Smaïl El Youssoufi, Said Salmi

**Affiliations:** 1 Anaesthesiology unit, Department of Obstetrics and Gynaecology, Ibn Rochd University Hospital, Casablanca, Morocco; 2 Department of Neurology, Ibn Rochd University Hospital, Casablanca, Morocco; 3 Department of Neurosurgery, Ibn Rochd University Hospital, Casablanca, Morocco

**Keywords:** Epidural neoplasms, central nervous system cavernous haemangioma, pregnancy, vascular surgical procedures

## Abstract

Cavernous haemangiomas are benign vascular malformations that can locate in the central nervous system. The epidural spinal location remains unusual. Pregnancy is known to be a precipitating factor. The aim of this study is to review general aspects of these lesions and specific facts about their relationship to pregnancy. A 32-year-old full-term pregnant woman is managed during early labor for a progressive spinal cord compression syndrome. After delivery, exploration by a lumbar MRI found an epidural vascular dorsal mass. Surgical exploration and histopathological examination confirmed the diagnosis of epidural cavernous haemangioma. The patient achieved complete recovery after 1 month. Spinal cavernous haemangiomas are rare malformations. Specific mechanisms seems to be involved in their growth during pregnancy. Although clinical and radiological presentation are spectacular and misleading, the prognosis is generally good, and urgent surgical treatment during pregnancy is usually not indicated.

## Introduction

Cavernous haemangiomas are benign vascular malformations that may locate in the central nervous system, especially in the brain, the cerebellum and the brainstem [1]. Spinal localisation is unusual, and represents 3 to 16% of spinal vascular malformations [2]. Epidural haemangiomas are particularly exceptional lesions. Indeed, from 1929 to 2006, only 80 cases of epidural cavernous haemangiomas have been reported in the literature [3]. Pregnancy is acknowledged to be a precipitating factor of this type of malformation, but the exact mechanism is obscure [4]. Their management during pregnancy also remains controversial. In the current report, we illustrate the course of this pathology through the case of a 32-year-old woman, whose cavernous haemangioma broke out in the third trimester of pregnancy. The aim of this report is to review general aspects of epidural cavernous haemangiomas and specific facts about their relationship to pregnancy.

## Patient and observation

We report the case of Mrs. D. F., 32 years old, living in rural areas, with a history of tuberculosis exposition (tuberculosis infection in stepfather), gravida 2 para 2, admitted to the obstetric emergency department for delivery of a full-term monitored pregnancy. The patient also reported weakness in her lower limbs gradually evolving for 2 months, confining her in wheelchair after 1 month of evolution. These symptoms had been neglected by the attending physician, who recommended the patient to consult for these symptoms after childbirth. The clinical examination on admission found a conscious patient, with stable haemodynamic and respiratory status, with T12 cord compression syndrome and a low back pain, without signs of radiculalgia. The gynaecological examination confirmed the labor progress. Given the neurological condition of the patient, being already in labor, and considering the unknown status of her spinal cord, a caesarean section was made under general anaesthesia without incident, allowing the extraction of a healthy newborn.

**Figure 1:**
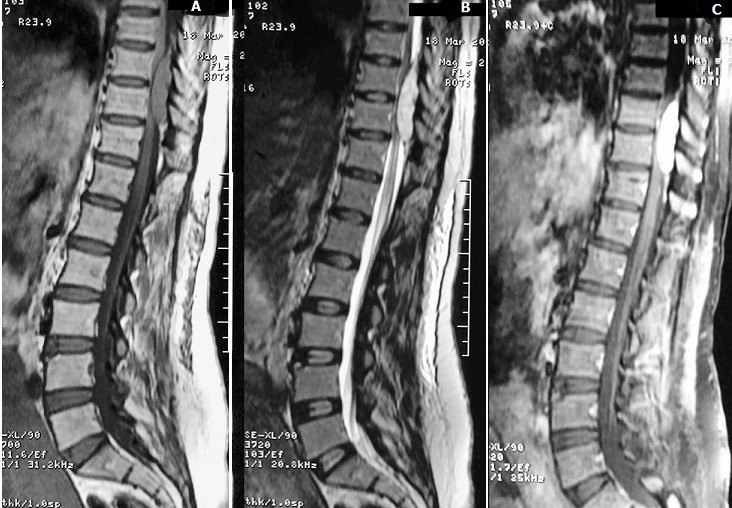
Sagittal unenhanced T1 weighted MR images: A) shows a homogeneous well-defined, ovoid epidural mass with isointense signal relative to the spinal cord, with a «wafting silk» sign. Sagittal T2-weighted images; B) shows a heterogeneous hyperintense epidural mass. Sagittal contrast-enhanced T1-weighted images; C) shows homogeneous intense enhancement of the mass

**Figure 2:**
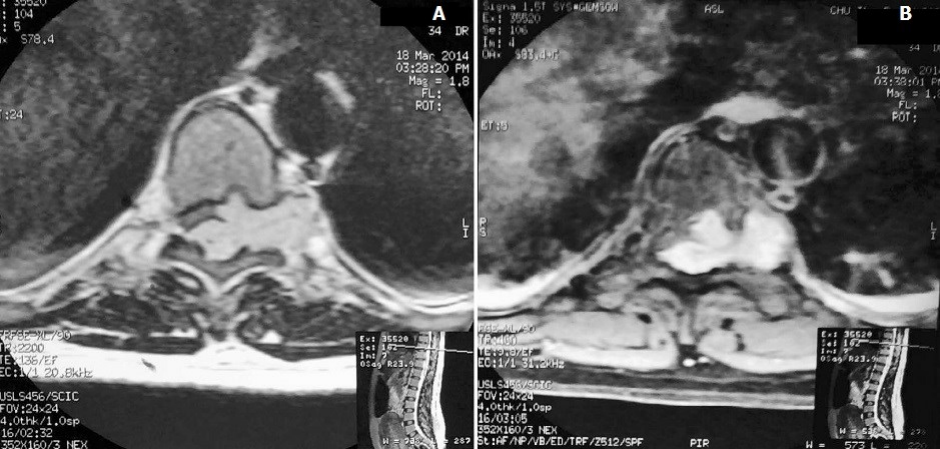
Transverse unenhanced: A) and contrast-enhanced; B) T1-weighted MR images show an epidural mass with homogenous intense enhancement, extending into and widening the left inter-vertebral foramen with a typical dumbell-shaped morphology and a bone erosion of vertebral body

The patient underwent a day after delivery a lumbar MRI which showed an well defined ovoid epidural mass extending from T9 to T10 with a lobulated contour realising a «wafting silk» sign (Figure 1), compressing and flattening the dorsal spine without infiltrating it, and widening the left foraminal region and eroding the body of the vertebrae with a dumbbell-shaped morphology in transverse images (Figure 2), compressing the nerve root. The mass showed isointense signal in T1-weighted images and hyperintense signal in T2-weighted images, with intense contrast enhancement.

**Figure 3:**
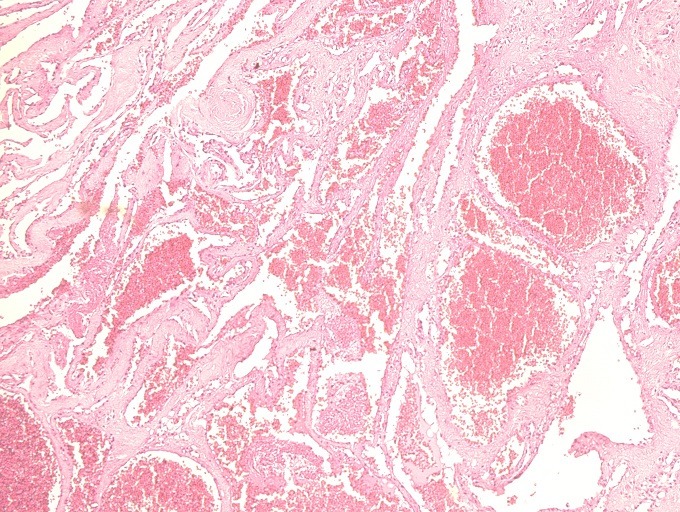
Image of the vascular tumour proliferation (eosin-hematoxylin staining; magnification × 20). Note the large vessel lumen, filled with red cells

The patient underwent surgery the next day. She was positioned in ventral decubitus. The neurosurgical exploration revealed a reddish vascular mass, bleeding on contact, measuring 30×24×13 mm, which was completely removed piece by piece after laminectomy, with good haemostasis achieved with Surgicel cellulose hemostats and bipolar coagulation. Histopathological examination of the mass confirmed its vascular origin, with many small cavernous vessels and thick hyalinised walls, without malignant cells (Figure 3). The post-operative outcome was uneventful. She started motor rehabilitation 3 days after the surgery. The patient was able to walk after 2 weeks. She achieved complete recovery after 1 month of follow-up.

## Discussion

Cavernous haemangiomas are vascular malformations that may occur in many organs. In the central nervous system, they can be intracranial or infrequently spinal [3]. These can be intramedullary, intradural, or vertebral epidural [5]. Pure epidural location, defined by the presence of 90% of the tumour volume in the epidural space [6], is unusual. Indeed, 80 cases have been reported in the literature from 1929 to 2006 [2], and represent 4% of epidural tumours and 12% of spinal haemangiomas [4]. They occur in 70% of cases in women [2]. The mean age vary between 30 and 60, with a peak occurring around 40 [3]. Consistent with that, a recent case series of 14 cases found a mean age of 51,64 years, although a male predominance was observed [7].

Cavernous haemangiomas are benign malformations, with slow development, classified by the predominance of vascular channel type (capillary, cavernous, arteriovenous, or venous) [4]. Their name is debated [1, 8]. Formerly called "cavernous haemangioma" or "cavernous malformations", the International Society for the Study of Vascular Anomalies adopted the new terminology of "venous malformations", according to the Mulliken and Glowek classification [9], but the above does not seem to make unanimity among neurosurgeons [8].

Even though comparable in pathological terms, there are some differences between the intraparenchymal and vertebral haemangiomas. The first ones seem to be poorly vascularised and occult on the angiographic level. As for the second ones, whether or not having an epidural extension, they are instead well vascularised with well individualised arterial afferents on angiography [10]. They often develop at the thoracic level, with a predilection for the T2-T6 segment and extend over two or more vertebral segments [11, 12]. The thoracic region has a poor vascular supply, resulting from a poor anastomotic network, in contrast to the regions above and below which are highly vascularised. Since the system of venous plexi draining the spinal cord is valveless, increased intrathoracic and intraabdominal pressure can result in venous engorgement, making a quiescent vascular tumour symptomatic, thus explaining the location of the symptomatic haemangiomas [13]. On the axial plane, they are usually found at the posterior part of the epidural space. This is probably due to the larger size of the posterior spaces [11]. Foraminal extension is also common, as has been observed in our patient, but it is usually isolated [10]. This foraminal extension was seen in 64% of patients in a case series, and it was hypothesised that it may be due to the loose tissue structure inside the neuroforamen [7].

Histologically, they are made of multiple and large sinusoidal spaces, joined together without interposed nervous or glial tissue. The wall of these vessels usually consists of a fibrohyalin capsule lined by a single endothelial lining without elastic or muscular frame [10]. The presence of a crown of macrophage cells with hemosiderin deposits in the periphery seems to be more specific of intramedullary haemangiomas [10].

Pregnancy is known to be a precipitating factor in the development of these malformations and their clinical expression. Several assumptions have been advanced to explain this relationship [14]: mechanical theory: by obstruction of the blood flow from the paravertebral veins into the inferior vena cava by the gravid uterus. This increase in venous pressure may lead to distension of the vascular channels in epidural haemangiomas and therefore a rapid increase in their size [4]; hormonal theory: by overexpression of angiogenic factors during embryogenesis, such as Vascular Endothelial Growth Factor, basic Fibroblast Growth Factor and Placental Growth Factor, leading to a significant increase in malformations during this period of pregnancy [15]. Estrogen would also play a role in the growth of these lesions, by acting directly upon the endothelium of the vascular channels [16].

This hormonal theory, combined with other mechanisms, such as the increase of the preexisting spinal cord ischaemia by pregnancy-associated hemodilution and anaemia, may also explain vascular accidents, such as thrombosis and haemorrhage, responsible for acute onset of symptoms [15]. However, we failed to found any evidence in the literature that these epidural lesions may declare acutely specifically during pregnancy. Trauma, physical activity and use of anticoagulants are also implicated in the aggravation of symptoms [7, 11]. Furthermore, the mechanical theory may explain the relatively acute onset of symptoms in the third trimester as the rapidly enlarging uterus cause increased intraabdominal and intrathoracic pressure [13]. Symptoms may resolve in early postpartum period, as a result of the rapid decrease in the pressure effect of the gravid uterus and the correction of the venous blood flow reversal [13].

Clinically, epidural cavernous haemangiomas seem to more frequently reveal by a gradual onset myelopathy, usually in less than a year [17], than radicular symptoms, probably due to a better capacity of nerve roots to tolerate long-term soft compression compared to the spinal cord [7]. This would explain why our patient did not show any radicular sign despite the important infiltration of the tumour through the neuroforamen. This mode of onset is comparable to the mode of presentation of spinal metastases [6]. Some authors have also reported an acute onset mode (11% to 21% of cases), secondary to a fast increase in lesion volume by haemorrhage or thrombosis [3, 7, 10]. This onset mode would depend on the location, growth rate, and biological behaviour of the malformation [18].

MRI is the most valuable method for preoperative diagnosis of spinal cavernous haemangiomas, and can provide information about localisation and extension of malformation [11]. They have a lobulated posterior shape, due to the soft texture of the tumour and the small size of the epidural space, realising a "wafting silk” sign. It looks like a spindle-shaped mass with two tapered ends, partially filling the posterior epidural space around the spinal cord [3]. It is generally isointense compared to the cord in T1-weighted images, and slightly hyperintense in T2-weighted images, with an intense homogeneous contrast enhancement after IV administration of gadolinium [6]. On the MRI axial sections, a "hourglass" or a "dumbbell" aspect is observed without important windening of the foramen [10]. A vertebral bone erosion is also possible as seen in our patient [3]. Intramedullary hyperintense images are possible in case of high degree cord compression due to tumour haemorrhage or fast growth [7]. Imaging characteristics in epidural haemangiomas are different from those of intramedullary hemangiomas[3]. Indeed, epidural hemangiomas are usually not enclosed in a hyposignal ring, which is the case for intramedullary hemangiomas. This is due to the easier elimination of peripheral hemosiderin outside the blood brain barrier due to a richer vascularity of epidural lesions [4, 7]. The differential diagnosis of this lesion arises with many spinal tumours including meningiomas, neurinomas, neurofibromas, schwanomas, metastases, angiolipomas, lymphomas, but also a herniated disc, haematoma [11], or spinal canal stenosis [19]. There are few data in the literature concerning the diagnostic value of angiography for cavernous haemangiomas, but it seems necessary unless a strongly evocative lesion is found on the MRI [10].

Surgical removal of the tumour is the standard procedure for these malformations [20]. Some authors reported the use of lateral position to clear the operating field, control vigorous intraoperative bleeding and reduce epidural venous pressure in the vertebral canal resulting from reduced chest and abdominal compression [7]. A total resection is recommended to avoid the risk of intraoperative bleeding [6]. For this, a microsurgical technique can be used in order to make the lesion free from its adhesions and from its feeder vessels. It is also recommended to shrunk progressively the haemangioma by bipolar coagulation starting at the part not in contact with the thecal sac [6, 10]. In our patient, an «en-bloc» resection was not possible despite the laminectomy, because of the important infiltration of the tumour and the bony erosion. Preoperative embolisation of the injury would contribute to reduce intraoperative bleeding [10].

However, the microsurgical removal would decrease the haemorrhagic risk and would make it controllable, making the usefulness of the technique questionable. Adjuvant radiotherapy for residual lesions is not recommended considering its side effect [20]. In this case, it is recommended to perform radiological monitoring of these lesions, and wait until their size allows for a second surgical extirpation in a further time [21].

Care of spinal cavernous haemangiomas during pregnancy rarely requires urgent surgical treatment. Most of the cases described in the literature were made following childbirth, by caesarean or vaginal delivery depending on obstetric conditions, with a good outcome [15].

Prognosis of epidural cavernous haemangiomas is generally good, with a recovery rate up to 92% [6], but would depend on the onset mode of the symptomatology [20]. Thus, a brutal onset would be correlated with a poor functional prognosis [17]. This onset mode might then be an indication for urgent surgery. Malignant degeneration was never reported [6].

## Conclusion

Spinal cavernous haemangiomas are rare malformations. This case supports the fact that pregnancy is probably a precipitating factor in their growth through specific mechanisms. Although clinical and radiological presentations are spectacular and misleading, the prognosis is generally good, and urgent surgical treatment during pregnancy is usually not indicated. However, these lesions require exploration and adequate monitoring to prevent any irreversible lesion.

## Competing interests

The authors declare no competing interests.
